# Intelligent Driving Assistant Based on Road Accident Risk Map Analysis and Vehicle Telemetry

**DOI:** 10.3390/s20061763

**Published:** 2020-03-22

**Authors:** José Terán, Loraine Navarro, Christian G. Quintero M., Mauricio Pardo

**Affiliations:** Department of Electrical and Electronics Engineering, Universidad del Norte, Barranquilla 081007, Colombia; mjteran@uninorte.edu.co (J.T.); lorainen@uninorte.edu.co (L.N.); mpardo@uninorte.edu.co (M.P.)

**Keywords:** driving assistant, driving diagnosis, accident risk maps, driving safety

## Abstract

Through the application of intelligent systems in driver assistance systems, the experience of traveling by road has become much more comfortable and safe. In this sense, this paper then reports the development of an intelligent driving assistant, based on vehicle telemetry and road accident risk map analysis, whose responsibility is to alert the driver in order to avoid risky situations that may cause traffic accidents. In performance evaluations using real cars in a real environment, the on-board intelligent assistant reproduced real-time audio-visual alerts according to information obtained from both telemetry and road accident risk map analysis. As a result, an intelligent assistance agent based on fuzzy reasoning was obtained, which supported the driver correctly in real-time according to the telemetry data, the vehicle environment and the principles of secure driving practices and transportation regulation laws. Experimental results and conclusions emphasizing the advantages of the proposed intelligent driving assistant in the improvement of the driving task are presented.

## 1. Introduction

Currently, road traffic accidents are one of the main causes of mortality according to the World Health Organization (WHO). Over 1.2 million people die every year on the world’s roads and millions more live with serious injuries or long-term adverse consequences. Traffic accidents are the ninth most common cause of death worldwide, and the main cause of death for young-adults between 15 and 29 years old. In Colombia, about 8000 people are victims of those accidents, including drivers, passengers, and pedestrians [1]. As a result, governments have taken preventive measures to mitigate the number of accidents, implementing traffic regulation laws considering risk factors (driving under alcohol influence or psychoactive substances, lack of driving skills, speeding, reckless drives, among others). However, these laws are not enough to cover the risks caused by bad driving practices. Therefore, over the years, intelligent driving assistance systems (I-DAS) have been created to mitigate incidents because most of the accidents frequently rely on driver performance [2]. Thus, these systems assist the driver in different tasks, such as getting oriented, increasing fuel-consumption efficiency, and proving useful information about both vehicle tracking and motion. The goal is to maintain the driver attention in the road and with that improve safety on the roads [3,4,5].

Although the term driving assistant can be very broad (due to the diversity of active and passive applications), the present paper emphasizes the concept of an expert advisor assistant that accompanies the driver while is operating a vehicle. The proposed system comprises on-board telemetry data and a road accident risk map analysis integrated jointly through fuzzy logic in an abstraction of driving regulations and secure driving techniques for real-time intelligent driving assistance. As commented, a real I-DAS employs telemetry and in-vehicle data acquisition for proper driving assistance. Vehicular telemetry systems focus on those variables that can be taken directly from the vehicle, such as the variation of yaw angle, pedaling, steering, speed, acceleration, and engine conditions, among others. Depending on how they have been implemented, these telemetry systems can be acquisition systems designed specifically for a particular vehicle, on board diagnostics (OBD) systems, or standard systems (adaptable telemetry devices), which can be used for different vehicles [6,7,8]. Vehicle telemetry data acquisition is an indispensable step before driving analysis. This allows observing the behavior of each monitored signal and identifying the relationship between these variables and the maneuver performed by the driver. 

At the same time, the road accident risk map analysis allows estimating the risk level of each road, so that it is possible to identify which roads are more dangerous than others. Consequently, it is most common to find road accident studies for a certain region or sector [9,10]. Here, the main goal is to identify the factors involved in road traffic accidents and analyze the relationship between them. The typical procedure in this type of work is to take a certain amount of accident data, analyze it through multiple statistical parameters; and then, organize and classify the accidents, roads, and sectors according to defined criteria (i.e., road accident risk maps). Thanks to the information obtained from the in-vehicle acquisition systems and the statistical studies of traffic accidents, different applications related to driving assistance can be developed. In this sense, the efficiency in the prevention of accidents depends significantly on the reliability of the collected data and the use of the appropriate methods of analysis. Today, the scientific community has taken the initiative in developing vehicular measurement devices, as well as tools that seek to assess driver performance. The aim is to establish all possible causes that lead to these accidents. Scientific research has mainly converged on the following topics: audiovisual records for the analysis of driving behavior (driver supervision, obstacle detection, proximity between vehicles, and pattern recognition for drowsiness, gaze tracking, road lanes or road signals) [11,12,13,14,15,16], driving modeling and driver behavior analysis (bad driving practices, careless or reckless driving) [17,18,19,20,21,22], driving style recognition (erratic driving assessment) [23,24].

In addition, intelligent systems applied in real vehicles have been motivated by the positive results obtained from simulators. The availability of resources and the multiple driving scenarios for experimentation (vehicle variables, types of road, types of vehicles, drivers, weather conditions, etc.) are the main advantages offered by them. In addition, they facilitate the study of new approaches in cases where implementing them directly in a real vehicle and a real environment can cause highly risky situations [19,25,26,27,28,29]. However, no matter how sophisticated the simulators are, they cannot provide all the physical aspects related to the actual driving process. Therefore, it is equally important to extend the studies to real scenarios [19]. 

In this context, the development of an intelligent driving assistant based on vehicular telemetry and road accident risk maps analysis in a real environment is proposed. The aim is to alert the driver by suggesting actions while the driving process is being carried out; therefore, careless situations can be avoided and with these, traffic accidents. While there have been studies that have developed intelligent driving assistants in real vehicles, as in the case of Yay et al. [5] and Kazuaki et al. [4], there are not many studies combining the road accident risk in the assistance process. This is particularly useful because, for example, some studies have shown that at higher speeds, the probability of road accidents becomes greater [1]. Nevertheless, it cannot be implied that speeding is a bad driving practice, because there are roads specially designed for high-speed driving and those not necessarily have high accident rates. At the same time, the acceleration and steering maneuvers are strongly related to the speed of the vehicle. However, what can be considered an aggressive steering maneuver at high speed may not be considered as such at low speed. This same concept, applied to the risk of accidents, implies that, for a road with a low accident rate, there would be no problem in performing maneuvers with a certain level of risk. This flexibility, when detecting and evaluating maneuvers, is one of the main goals in the development of this proposed intelligent driving assistant. 

## 2. Method

**Figure 1:**
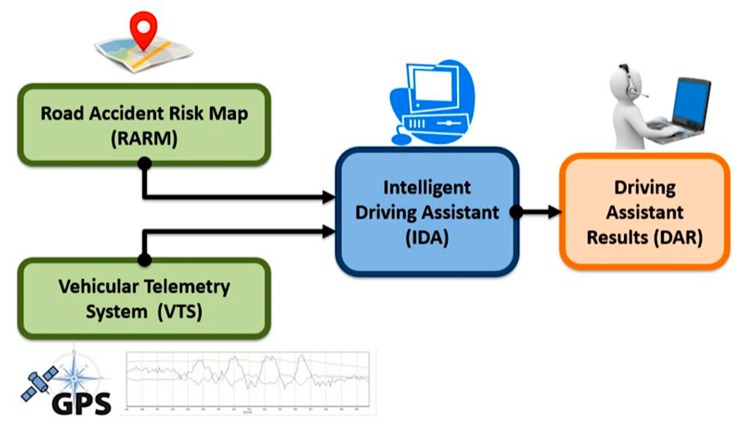
Intelligent Driving Assistant. System scheme.

The implementation of an intelligent driving assistant in a real environment looks for generating alerts to support the driver in real-time. Figure 1 shows the system components of the proposed assistant. This architecture considers the expert knowledge abstraction of driving laws and secure driving practices (IDA), along with the data related to the vehicle motion state (VTS) and its surroundings (RARM).

### 2.1. Road Accident Risk Map (RARM)

The risk maps are usually constructed by considering the statistics related to accidents in a specific area or road. According to the manual “roadway safety information analysis” [30], the crash rate by roadway mileage is an assessment parameter used to identify which roads present a higher risk than others. Equation 1 allows the calculation of this rate as the road accident risk (*RAR*),

(1) $$RAR=\frac{C}{N\times L}.$$

Building the RARM involves first calculating the *RAR* of each road (length *L* in kilometers), for a number of accidents (*C*) in a period of time (*N*). A database is produced with the resulting information, and is incorporated into the driving assistant. The concept of “tags” is used to classify the roads. Roads are categorized into three types according to their function as [31,32]: “highway”, designed for high-speed traffic flow, “urban”, designed to deliver traffic to highways (in-city roads), and “local” designed for low-speed traffic flow.

### 2.2. Vehicular Telemetry System (VTS)

**Figure 2:**
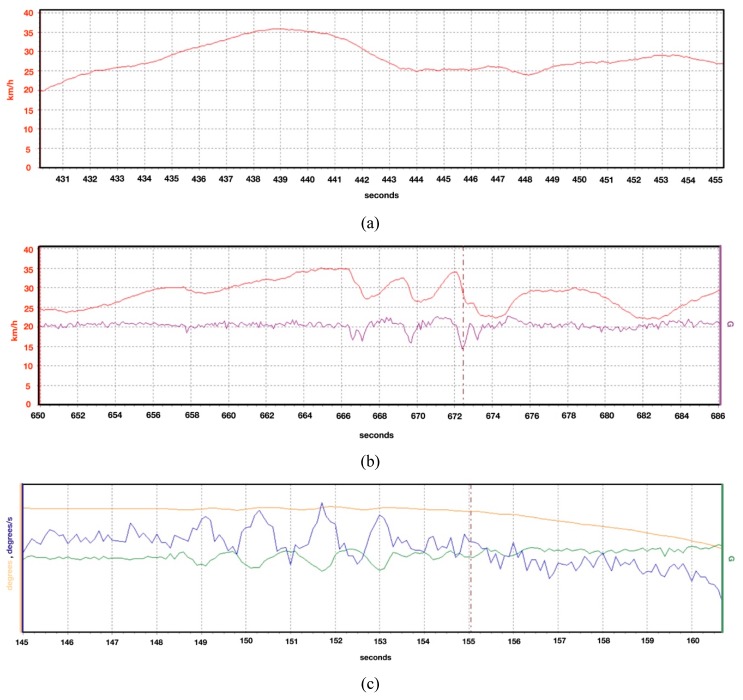
VTS signals: (**a**) Speed (red); (**b**) Speed (red) and Longitudinal Acceleration (purple); and (**c**) Heading (orange), Yaw Angle Rate (blue) and Lateral Acceleration (green).

The vehicular telemetry system consists of an electronic device easily adaptable to any car. It is responsible for monitoring variables related to the vehicle movement (driver maneuvers), and also for recording inside and outside the vehicle using video cameras to supervise the driving process. The data (signals) acquired from the VTS are geo-referenced via GPS standard protocol (NMEA standard). When the signals are acquired, they are continuously processed by the vehicle on-board computational system to provide real-time assistance. In this work, three risky maneuvers are categorized:

- Speeding: corresponds to those time instants in which the driver exceeded the speed limit allowed on the road, so it could be directly determined by observing the vehicle “speed” [km/h] (SPD). In Colombia, the maximum SPD is 40 km/h for local roads, 60 km/h for urban roads and 90 km/h for highways [33,34]. Figure 2a shows an example for local roads of how speed behaves in time.
- Bad pedaling: corresponds to the incorrect pedal handling (throttle and brake), when a driver performs abrupt or sudden acceleration/deceleration actions. It can be directly determined by observing the vehicle “longitudinal acceleration” [G] (LA). Best practitioners establish that the acceleration process should be progressive over time; which represents values between 0.1 G and 0.23 G in magnitude [35]. A maximum LA of 0.23 G for local roads, and 0.17 G for urban and highways is chosen. Figure 2b shows an example for local roads of how longitudinal acceleration behaves in time along with speed. It can be seen that, for abrupt deceleration maneuvers, the longitudinal acceleration perceives such variation, whereas for gradual decelerations it does not.
- Bad steering: corresponds to the incorrect handling of the steering shaft (also known as the steering column), when the driver performs abrupt or sudden turning maneuvers (changes in orientation). It can be determined by observing, the “heading” [°], the “yaw angle rate” [°/s] (YAR) or the “lateral acceleration” [G] of the vehicle, however, it is decided to use only the YAR as a measure of bad steering. A low YAR magnitude means normal use and not dangerous driving. YAR values close to 30 °/s are considered to be sharp fluctuations, and hence aggressive steering maneuvers [36,37]. In this study, a fixed maximum YAR value of 27.5°/s for local roads and 21°/s for urban and highways is used. Figure 2c shows a sample of these signals for local roads. The yaw angle rate presented the largest range of variation, being the most sensitive signal to detect steering maneuvers.

**Figure 3:**
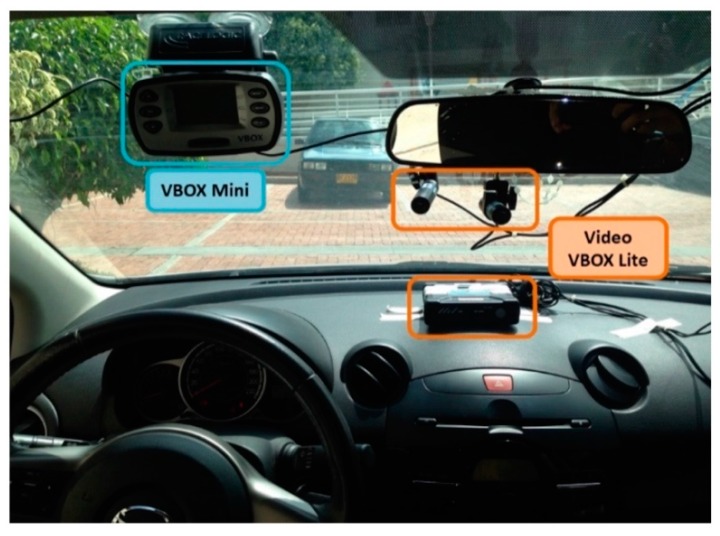
Vehicular Telemetry System (VTS).

The RACELOGIC VBOX, a device based on GPS, is a high precision performance meter used in real vehicles for automotive testing and provides considerable data related to the vehicle motion state [38]. Thus, this device is adopted as the VTS. Figure 3 shows the VBOX equipment.

### 2.3. Intelligent Driving Assistant (IDA)

The proposed approach implements fuzzy logic in the design of the intelligent agent, due to the advantages provided related to adaptable evaluation criteria and similarity to human reasoning. Such an approach is particularly suitable since the goal is to develop an intelligent agent that approximates the assessment that an expert driver would make concerning driving maneuvers.

**Figure 4:**
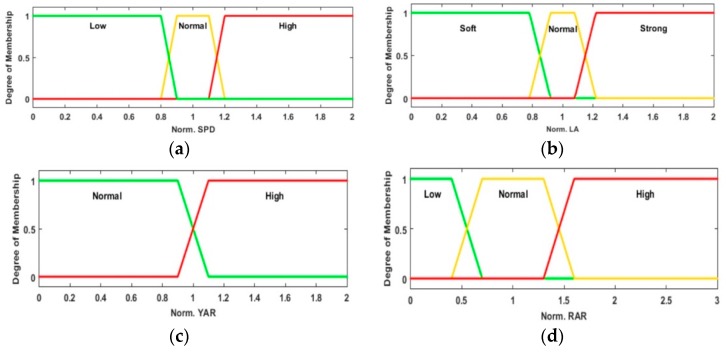
Proposed input variables membership functions: (**a**) Normalized Speed (*SPD_Norm_*), (**b**) Normalized Long. Acceleration (*LA_Norm_*), (**c**) Normalized Yaw Angle Rate (*YAR_Norm_*) and (**d**) Normalized Road Accident Risk (*RAR_Norm_*).

**Figure 5:**
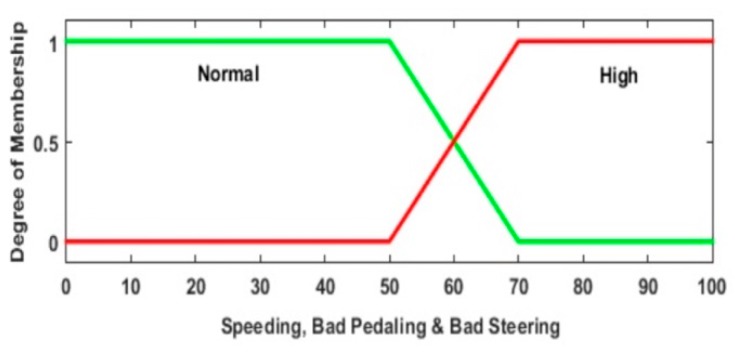
Proposed output variables membership functions: *Speeding*, *Bad Pedaling* and *Bad Steering*.

The abstraction of the expert knowledge, based on traffic regulation laws and secure driving practices, is mapped in a set of Mamdani fuzzy inference rules, along with the membership functions of the input and output variables. For signal analysis, it is decided to process the normalized value of the input variables, instead of the variable directly. This normalization is done based on the limits allowed for each signal. The main reason to employ this standardization is that the approach is to implement a single intelligent assistant adaptable to different roads by updating the maximum limit value for a specific road, instead of implementing an intelligent assistant for each road type. The agent processes four input signals. Figure 4 shows their membership functions. The defuzzifying process allows getting a magnitude (by centroid of the area) in order to determine which driving assistance is emitted. Figure 5 shows the three fuzzy variables (outputs) for risky maneuvers: speeding, bad pedaling, and bad steering. The same function set is used for each maneuver.

**Figure 6:**
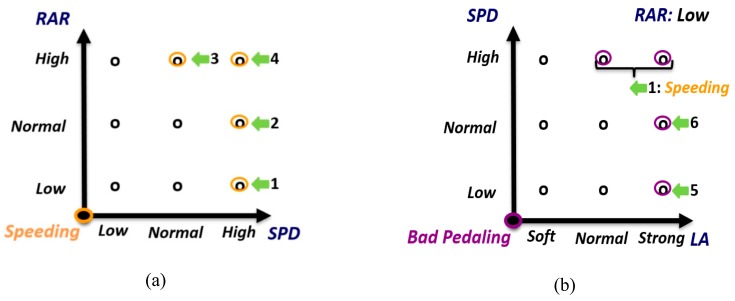

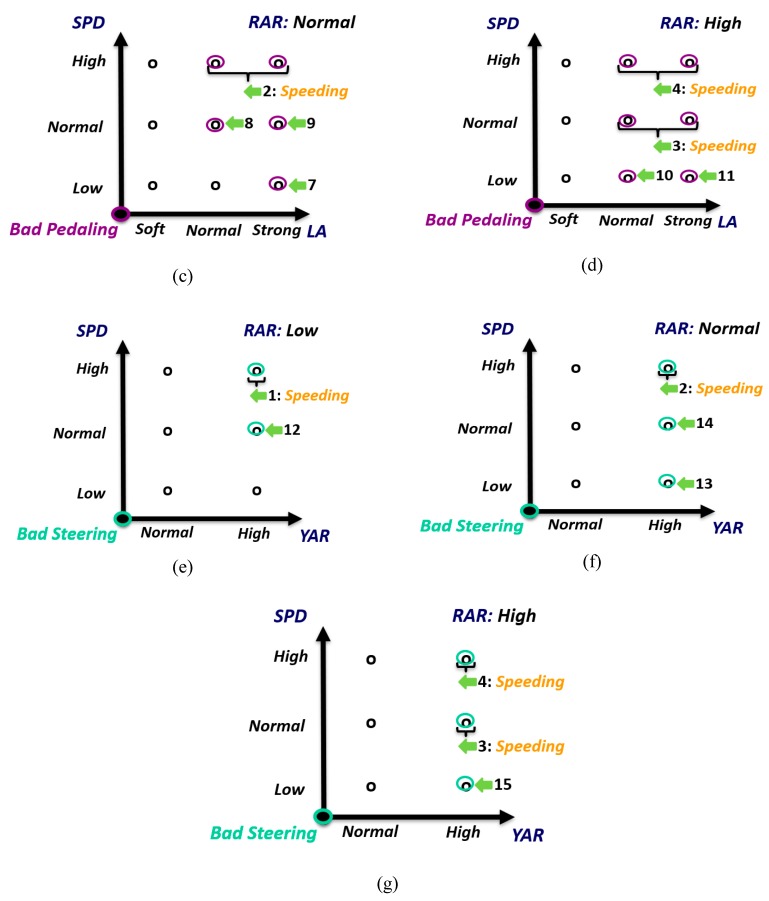
Fuzzy rule set selection process. (**a**) *Speeding* detection; (**b**), (**c**), (**d**) *Bad Pedaling* detection; and (**e**), (**f**), (**g**) *Bad Steering* detection.

Then, the next step is the association of the previous fuzzy variables. The selection process of the fuzzy rule set is made through the schemes shown in Figure 6. The detection of risky maneuvers is based on the following associations: speeding depending on speed (SPD) and road accident risk (RAR); bad pedaling depending on longitudinal acceleration (LA), SPD, and RAR; and bad steering depending on yaw angle rate (YAR), SPD, and RAR. The structure of the diagrams in Figure 6 consists of locating the input variables on the X- and Y-axes, and the output variable on the Z-axis (out of the paper). The dynamics for the rule selection are presented below.

First, the rules to detect the speeding maneuver are selected (see Figure 6a). Speeding is defined in these circumstances: (1) “high” SPD and “low” RAR; (2) “high” SPD and “normal” RAR; (3) “normal” SPD and “high” RAR; and (4) “high” SPD and “high” RAR. The selected circumstances produce Rules 1, 2, 3 and 4, respectively.

Then, the rules to detect bad pedaling are selected (see Figure 6b–d for the different RARs). For cases with “low” RAR (Figure 6b), bad pedaling is defined in these circumstances: (5) “strong” LA and “low” SPD; and (6) “strong” LA and “normal” SPD. The selected circumstances produce Rules 5 and 6, respectively. Since cases with “high” SPD are already covered by Rule 1 (speeding detection), they are not considered. The same procedure is followed in the cases of “normal” RAR for Rules 7, 8 and 9 (Figure 6c), and “high” RAR for Rules 10 and 11 (Figure 6d).

Similarly, the rules to detect bad steering are shown in Figure 6e, f and g for the different RARs. For “Low” RAR (Figure 6e), bad steering is defined in these circumstances: (12) “high” YAR and “normal” SPD, giving rule 12. Since the case with “high” SPD is already covered by Rule 1 (speeding detection), it is not considered. The same procedure is followed for “normal” RAR (Figure 6f), giving Rules 13 and 14; and “high” RAR (Figure 6g), giving Rule 15.

According to these selection dynamics, the speeding maneuver is the most important to detect (hierarchically). The cases of bad pedaling and bad steering, which are not considered because of the speeding detection, are situations that are not contemplated due to the extreme risk involved, and it would most likely result in traffic accidents.

Table 1 presents the proposed rules set for the fuzzy inference system.

### 2.4. Driving Assistant Results (DAR)

**Figure 7:**
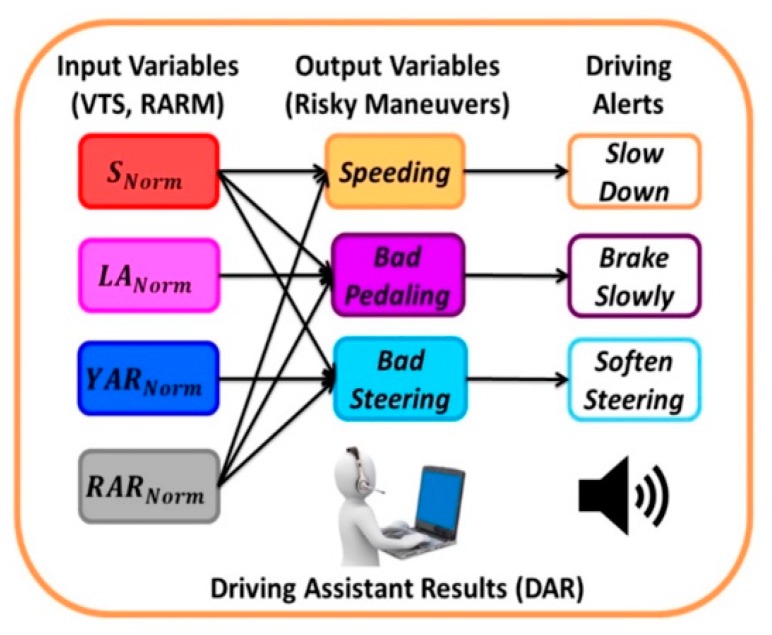
Driving assistant alerts.

Figure 7 shows the variables dependency followed by the fuzzy rules (Table 1) and the driving assistant alerts. The driving advice is set for each detected maneuver, generating a total of three alerts. The established alerts are: “slow down”, “brake slowly” and “soften steering”. The system issued advice in the order shown in Figure 7, if more than one maneuver was detected.

### 2.5. Developed Computational System 

**Figure 8:**
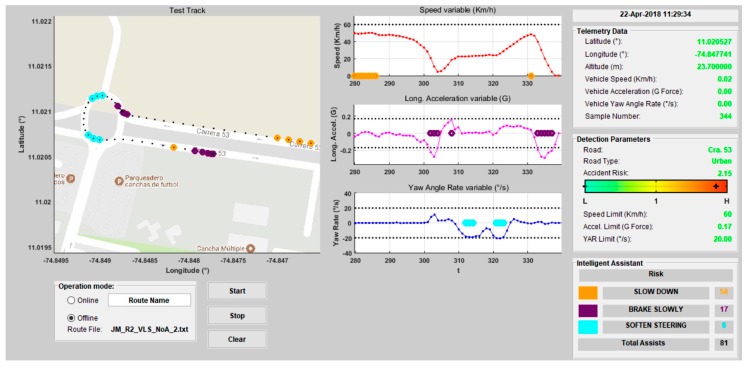
Software interface for Intelligent Driving Assistant (IDA).

The developed computational system allows two types of analysis to be performed: online analysis for evaluation in real-time, and offline analysis to evaluate driving registers in a post-driving analysis. Therefore, a software interface is developed to achieve friendly user interaction. Here, all the information handled by the assistant (vehicle motion state, environment, and intelligent assistance) is presented for both types of analysis. Figure 8 shows the interface designed for the computational system.

## 3. Experimental Results and Discussion 

The results presented in this section illustrate the proper functionality of the intelligent assistant in a real environment, as well as how the abstraction of expert knowledge is correctly applied in the driving maneuvers assessment. Three types of tests are made to quantitatively assess the performance of the IDA. The first one consists of a functionality test, the second one verified its effectiveness, and the third one seeks to evaluate the incidence of the use of the assistant in the driving process. It is important to note that if none of the considered risky maneuvers are detected; then, the IDA concludes an acceptable driving practice, and the system does not generate any alert (assistance). 

Before presenting the test results, the parameters and road scenarios in which they are carried out are discussed.

### 3.1. Road Scenarios

**Figure 9:**
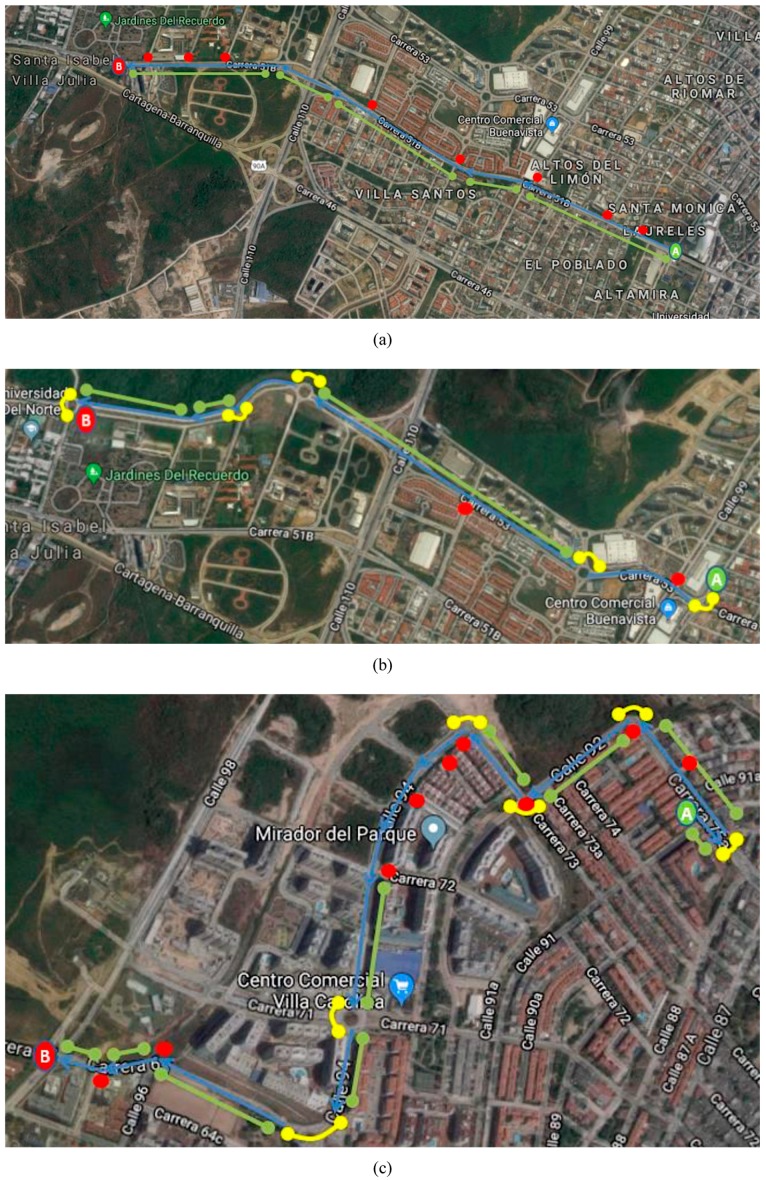
Types of Road (A: Start point, B: End point): (**a**) Route 1, (**b**) Route 2 and (**c**) Route 3.

The system developed in this study could adapt to driving environments and is also suitable for any vehicle. It is desirable to demonstrate the environmental adaptability with different road types, therefore a total of three routes are selected. Such selection is made based on the characteristics of the three types of road infrastructure: straight sections, curved sections, and intersections. Figure 9 shows the routes and Table 2, their characteristics. It is also important to demonstrate compatibility and system management with different types of vehicles. Hence, the vehicle weight and dimensions are used as classification criteria, and two types of vehicle are used:

- Light vehicles (*V_1_*): Those with a weight of less than 1000 kg. This category includes short and small vehicles of the hatchback type.
- Heavy vehicles (*V_2_*): Those with a weight greater than 1000 kg. This category includes long and large vehicles of sedan type.

### 3.2. Functionality Test

**Figure 10:**
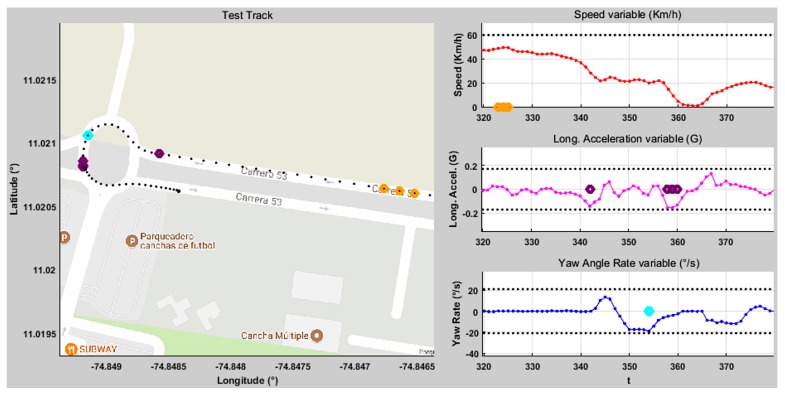
End of *Route 2* for driver D_1_ assistance results.

**Figure 11:**
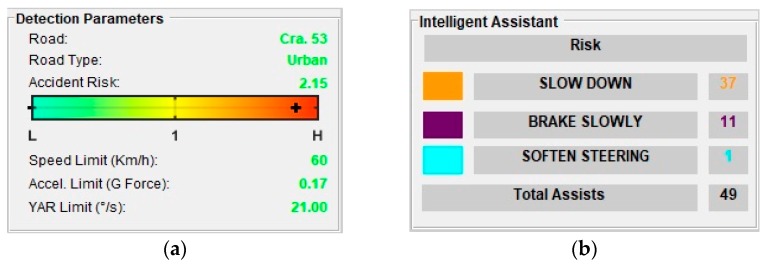
Route 2: (**a**) Assessment parameters, (**b**) Assistance results.

The purpose of the functionality test is assuring that the system detects risky maneuvers and issues alerts coherently, according to the behavior of the monitored variables (SPD, LA, YAR) and the environment (RAR). The following example illustrates the system operation on one of the routes (Figure 10 and Figure 11).

The assessment parameters remain constant throughout the trip, as Route 2 is always on the same road section. Figure 10 shows the end of Route 2, and Figure 11a presents the assessment parameters for this route. The alerts given are three speeding alerts (orange marks), four bad pedaling alerts (purple marks), and one bad steering alert (cyan marks). The vehicle is on a road with a “high” RAR in each of these cases. speeding alerts are given when a speed of 49.47 km/h is reached (which corresponds to a “low-normal” SPD level) activating fuzzy Rule 3 (Table 1). For bad pedaling, alerts are given if accelerations up to −0.15 G (braking) are reached (“normal” LA) at speeds of approximately 28 km/h (“low” SPD) activating Rule 10. Bad steering alerts are given when a turning of −19°/s (left turn, “normal-high” YAR) is reached at a speed of 20 km/h (“low” SPD) activating Rule 15. Figure 11b shows the total assists/risky maneuvers obtained on Route 2.

This rational behavior is consistently exhibited on the other routes, suggesting that the intelligent assistant works properly and fulfills its task of assisting the driver real-time during the driving process.

### 3.3. Efficiency Test

An efficiency test is carried out to determine the overall effectiveness of the driving assistant. The methodology consists of taking a set of driving samples (three routes, two vehicles, eight drivers, and three repetitions per driver in each scenario, making a total of 144 experiments), and applying to each one the analysis made by the IDA. The objective is to compare the number of correct alerts versus the total number of alerts issued. The average correct alerts provided by the driving assistant is adopted as the assessment metric.

**Figure 12:**
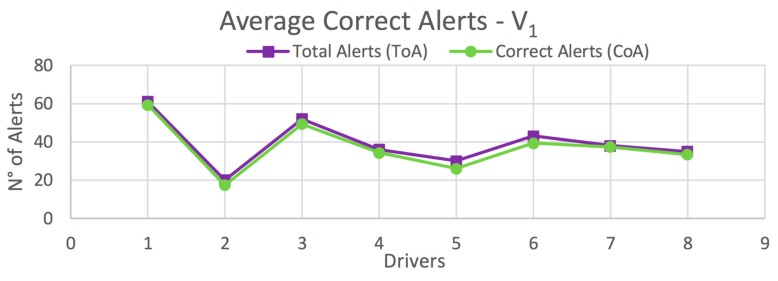
Average total correct alerts on all routes for *V_1_* (Light Vehicle).

**Figure 13:**
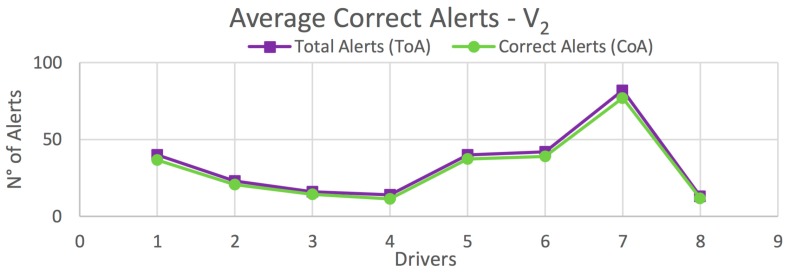
Average total correct alerts on all routes for *V_2_* (*Heavy Vehicle*).

**Figure 14:**
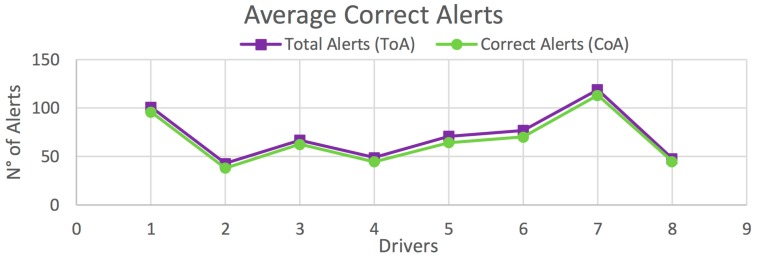
Average total correct alerts on all routes for all vehicles (*V_1_ & V_2_*).

The results obtained in this test are organized according to the type of vehicle. Figure 12 shows the average correct alerts obtained for each driver on all routes for vehicle *V_1_*. Table 3 presents the percentage of correct alerts obtained for each driver from Figure 12, as well as those obtained on each route separately. Similar results are shown for vehicle *V_2_* in Figure 13 and Table 4, and for the global case in Figure 14 and Table 5.

In the case of *V_1_*, high percentages of correct alerts (77.5%, 91.6%, 97.5%) are obtained for Routes 1–3, respectively; and a total of 93.2% for all drivers on all routes (Table 3). In the case of *V_2_*, the percentages of correct alerts are 74%, 86.7%, 96.7% for Routes 1–3, respectively; and a total of 90.2% for all drivers on all routes (Table 4). This results in a global average of 92.4% of correct alerts (Table 5). In most cases, and also in the global case, a high percentage of correct alerts being issued is observed. Thus, it is suggested a high efficiency in the detection of risky maneuvers, and consequently, in providing driving assistance.

### 3.4. Driving Performance Test 

The purpose of this test is to evaluate the incidence of using the assistant in the driving process. The methodology consists of taking driving journeys (the same as those used in the efficiency test), but this time for two cases: with and without the assistant (making a total of 288 experiments). The evaluation metric is the average decrement in the number of risky maneuvers. First, the routes are driven with the assistance system in operation but disabling the audio alerts. Hence, the risky maneuvers detected are still recorded. Later, the same route is driven again but with the audio alerts on. It should be noted that, although the assistant is responsible for issuing driving advice, it is the driver responsibility to follow or ignore the assistance. The goal is to compare the number of risky maneuvers/alerts obtained with and without assistance.

**Figure 15:**
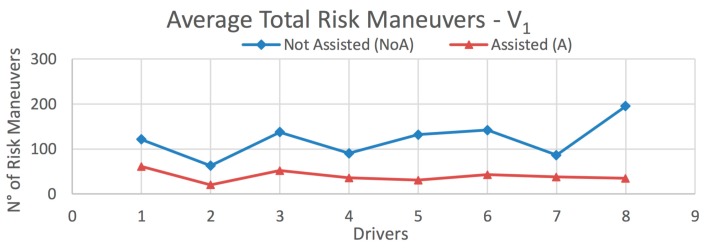
Average total risk maneuvers on all routes for *V_1_* (Light Vehicle).

**Figure 16:**
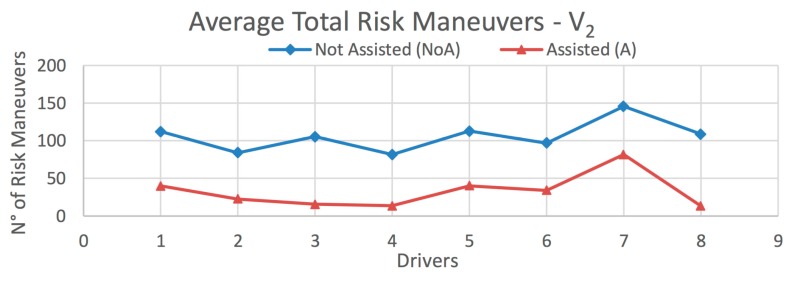
Average total risky maneuvers on all routes for *V_2_* (*Heavy Vehicle*).

**Figure 17:**
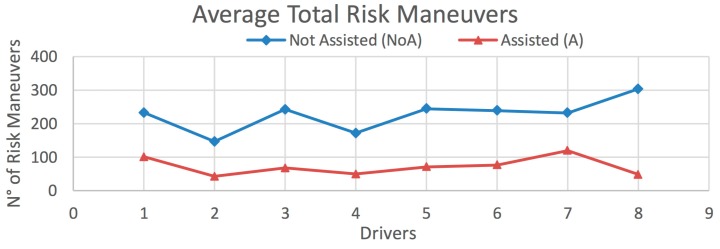
Average total risky maneuvers on all routes for all vehicles (*V_1_* & *V_2_*).

The results obtained from this test are presented in Figure 15 and Figure 16, and Table 6 and Table 7, according to the type of vehicle. Figure 15 shows the average total number of risky maneuvers made by each driver on all routes for vehicle *V_1_*. Table 6 presents the decrement in the percentage of risky maneuvers for each driver, as well as those obtained on each route separately. Analogous data for vehicle *V_2_* are shown in Figure 16 and Table 7, and for the global case in Figure 17 and Table 8.

In the case of vehicle *V_1_*, the percentage decrement in risky maneuvers is 35%, 55.9%, and 71.6% for Routes 1–3, respectively; and a total of 65.7% for all routes (Table 6). The corresponding results for vehicle *V_2_* are 43.1%, 73.2%, and 67.5%, for Routes 1–3, respectively: and 70.8% for all routes (Table 7). The decrement in risky maneuvers for all vehicles over all routes is 67.8% (Table 8). In most cases, the number of risky maneuvers decreases after driving the routes with the IDA assistance enabled. This suggests that there is a positive influence of using the assistant.

## 4. Statistical Validation

Statistical analysis is carried out to verify that the results obtained from the selected samples are representative of other drivers in the population. In the current work, this statistical validation is carried out through hypothesis testing, in which a null hypothesis (*H_0_*) and an alternative hypothesis (*H_1_*) are proposed. According to the structure of this type of test, the term *H_0_* corresponds to the hypothesis tested with the goal to be rejected leading to the acceptance of hypothesis *H_1_*. *H_1_* usually corresponds to the question or theory that is desired and opposite to *H_0_* [35].

In the case of the efficiency test, the following hypotheses are established:

- Null Hypothesis (*H_0_*): The average percentage of correct alerts is less than or equal to 90% (*µ ≤ 0.90*).
- Alternative Hypothesis (*H_1_*): The average percentage of correct alerts is greater than 90% (*µ > 0.90*).

It is decided to use the right tail of the t-student distribution to determine the veracity of *H_0_* with a confidence level of 95% (*1* − *α = 0.95*), considering the hypothesis and the number of samples (repetitions per driver) in this test. Equation (2) describes the calculation of the statistical value t_α_ to evaluate *H_0_* [35]:

(2) $$t_{\propto }=\frac{\bar{X}-\mu }{S\;/\;\sqrt{N}}$$

where *t_α_* is the distribution value for a certain level of significance (*α*), and a certain degree of freedom (*v = N – 1*), $\bar{X}$ is the sample mean, *µ* is the population mean, *S* is the sample standard deviation, and *N* is the number of samples. Rearranging Equation (2) gives Equation (3):

(3) $$\mu =\bar{X}-\left(t_{\alpha }\;*\;S\;/\;\sqrt{N}\right)$$

(4) $$\mu =0.924-\left(1.714\;*\;0.024\;/\;\sqrt{24}\right)=0.92$$

Using the following values obtained from the experimental results (*N = 24,* *α = 0.05, v = N − 1 = 23, t_0.05_ = 1.714,* $\bar{X}$*= 0.924* and *S = 0.024*), Equation (4) produce a *μ > 0.9* (Table 5). Thus, the rejection of *H_0_* is implied, and therefore the veracity of *H_1_*, allowing the validation of these results for other drivers.

For the driving performance test, the following hypotheses are established:

- Null Hypothesis (*H_0_*): The average percentage decrease in risky maneuvers is less than or equal to 50% (*µ ≤ 0.5*).
- Alternative Hypothesis (*H_1_*): The average percentage decrease in risky maneuvers is greater than 50% (*µ > 0.5*).

Similarly, as in the previous analysis, Equation (3) is used to verify *H_0_* with a confidence level of 95% (*1 –* *α = 0.95*). Using the following values obtained from the experimental results (*N = 24,* *α = 0.05, v = N − 1 = 23, t_0.05_ = 1.714,* $\bar{X}$*= 0.678* and *S = 0.108*) (Table 8), Equation (5) becomes:

(5) $$\mu =0.678-\left(1.714\;*\;0.108\;/\;\sqrt{24}\right)=0.64$$

As *µ* > 0.5 implying the rejection of *H*_0_, and therefore the veracity of *H*_1_. This allows the validation of these results for other drivers.

## 5. Conclusions

In this work, an intelligent driving assistant based on road accident risk map analysis and vehicle telemetry is implemented in a real environment. The results demonstrate the relevance of the design and implementation of vehicle safety systems within the intelligent transport system (ITS) framework. An intelligent agent capable of assisting the driver in situations of driver carelessness is developed. The present fuzzy-logic-based IDA becomes a useful support element for the driver during the driving process. This IDA provides suggestions when risky maneuvers are detected for different road scenarios. The driving assistant uses an adaptive evaluation criterion that allows proper detection of risky maneuvers, based on information related to the VTS, the RARM, and supervision by video recording.

The performance of the system is evaluated in three tests. The first consists of a functionality test, which verifies that the assistant issued coherent driving alerts when the user is at risk by performing an established risky maneuver in real-time (system proper operation). The second consists of an efficiency test, which compares the number of correct alerts versus the number of total alerts issued. The system achieves an efficiency above 90% for correct alerts for the test routes. This suggests an optimal behavior within the ITS framework. Finally, the third test evaluates the influence of the assistant on driver performance comparing the number of risk maneuvers performed with the driving assistance enabled and disabled. A decrement in the number of risky maneuvers above 50% is observed in most cases, considering the variables, factors, road scenarios, and driver behavior. This demonstrates a positive influence of using the assistant and shows the importance of driver behavior in improving road safety [4,5]. In addition, statistical validation (confidence level of 95%) is carried out to verify that the results obtained for the driving study in a real environment, are equally valid for other drivers (population) [39].

The adaptability of the intelligent assistant for different types of vehicles and different types of roads is one of the most important aspects to consider for real environments. To address this, two vehicles are used and three routes are selected for the experiments. The vehicle dimensions and weights and the route characteristics (straights, curves, and intersections) are considered. The consistent behavior of the results shows that the adaptability requirement is met due to the telemetry system characteristics (an easily adaptable device for any car) and the constant updating of the assessment parameters according to the vehicle location (adaptive evaluation criteria for each type of road). In all experiments, it is assumed that the driver behaved rationally and followed the recommendations provided by the assistant.

As an application in a real environment, based on the analysis of the results, it is possible to establish future improvements and propose future studies to continue this line of research. One of the most important aspects to study is the elimination of false warnings. Although a high efficiency in issuing correct alerts is achieved, there is still the possibility of eliminating false alerts entirely. The false alerts occur because of the inaccuracy of the data received from the VTS (due to the interruption of the satellite signal by external factors). Possible countermeasures are to opt for an Inertial Navigation System, as a support for the GPS signal (INS GPS integration), which enables tracking of the vehicle to be maintained during those short periods of time in which the satellite signal is disabled (hardware alternative) [40], or opt for a statistical treatment of the GPS signal (software alternative), to mitigate the lack of precision at those moments of time [41].

Another aspect to improve is the development of a higher resolution accident risk map, that is, not only having the risk of an accident by road section, but by sub-section or intersection, therefore increasing the adaptability of the assistant for the different road scenarios [30]. Similarly, an alternative method to study the influence of the assistant on the driver, it is proposed to use other assessment criteria, from that used in this study (number of risk maneuvers performed), to evaluate driving performance.

It is expected that, in the future, as new technologies emerge, these auxiliary schemes will continue in process of improvement and that most commercial cars (even connected autonomous vehicles) will have such intelligent driving assistance systems implemented.

Finally, the exploration of other computational intelligence techniques to develop new assessment approaches for intelligent driving assistants in the framework of ITS is desirable.

## Figures and Tables

**Table 1:** Proposed inference rules for the Intelligent Driving Assistant (IDA).

Rule	Input				Output		
	Norm SPD	Norm LA	Norm YAR	Norm RAR	Speeding	Bad Pedaling	Bad Steering
**1**	High			Low	High		
**2**	High			Normal	High		
**3**	Normal			High	High		
**4**	High			High	High		
**5**	Low	Strong		Low		High	
**6**	Normal	Strong		Low		High	
**7**	Low	Strong		Normal		High	
**8**	Normal	Normal		Normal		High	
**9**	Normal	Strong		Normal		High	
**10**	Low	Normal		High		High	
**11**	Low	Strong		High		High	
**12**	Normal		High	Low			High
**13**	Low		High	Normal			High
**14**	Normal		High	Normal			High
**15**	Low		High	High			High

**Table 2:** Road details: Route 1, Route 2 and Route 3.

Route 1		
	Approx. Distance	3.5 km
	Approx. Number of Straights	5 (long straights)
	Approx. Number of Curves	0
	Approx. Number of Intersections	8
**Route 2**		
	Approx. Distance	3 km
	Approx. Number of Straights	3
	Approx. Number of Curves	5
	Approx. Number of Intersections	2
**Route 3**		
	Approx. Distance	1.76 km
	Approx. Number of Straights	9
	Approx. Number of Curves	6
	Approx. Number of Intersections	9

**Table 3:** Percentage of correct alerts for *V_1_* (Light Vehicle): Route 1, Route 2, Route 3 and all routes.

Percentage of Correct Alerts [%]									
Drivers Routes	D_1_	D_2_	D_3_	D_4_	D_5_	D_6_	D_7_	D_8_	Avg.
**Route 1**	77.8	66.7	75.3	88.9	66.7	88.9	88.9	66.7	77.5
**Route 2**	97.8	84.6	91.7	94.4	79.2	92.6	100	92.6	91.6
**Route 3**	100	100	98.9	100	100	83.3	97.6	100	97.5
**All Routes**	97.3	86.7	94.9	95.4	86.7	91.5	98.2	95.2	93.2

**Table 4:** Percentage of correct alerts for *V_2_* (Heavy Vehicle): Route 1, Route 2, Route 3 and all routes.

Percentage of Correct Alerts [%]									
Drivers Routes	D_1_	D_2_	D_3_	D_4_	D_5_	D_6_	D_7_	D_8_	Avg.
**Route 1**	50.0	55.6	66.7	66.7	83.3	83.3	86.7	100	74.0
**Route 2**	86.3	83.3	87.5	83.3	92.3	87.3	90.2	83.3	86.7
**Route 3**	100	100	95.2	83.3	97.2	100	97.6	100	96.7
**All Routes**	91.7	89.9	89.6	81.0	93.3	92.9	93.9	89.7	90.2

**Table 5:** Percentage of correct alerts on all routes for all vehicles (*V_1_ & V_2_*).

Percentage of Correct Alerts [%]										
Drivers	D_1_	D_2_	D_3_	D_4_	D_5_	D_6_	D_7_	D_8_	Avg.	Std. Dev.
**All Routes**	95.0	88.4	93.5	91.2	90.6	91.3	95.2	93.8	92.4	2.4

**Table 6:** Decrement percentage of risky maneuvers for *V_1_* (Light Vehicle): Route 1, Route 2, Route 3 and all routes.

Percentage of Correct Alerts [%]									
Drivers Routes	D_1_	D_2_	D_3_	D_4_	D_5_	D_6_	D_7_	D_8_	Avg.
**Route 1**	33.3	40.0	33.3	30.0	78.6	11.1	11.1	42.9	32.0
**Route 2**	38.2	15.2	77.6	55.8	77.3	52.8	43.1	87.4	55.9
**Route 3**	71.4	88.6	32.3	71.6	76.3	93.6	69.3	69.9	71.6
**All Routes**	49.6	68.1	62.3	60.4	77.0	70.0	56.2	82.2	65.7

**Table 7:** Decrement percentage of risky maneuvers for *V_2_* (Heavy Vehicle): Route 1, Route 2, Route 3 and all routes.

Percentage of Correct Alerts [%]									
Drivers Routes	D_1_	D_2_	D_3_	D_4_	D_5_	D_6_	D_7_	D_8_	Avg.
**Route 1**	50.0	0.0	0.0	12.5	80.0	60.0	67.4	75.0	43.1
**Route 2**	70.0	85.7	86.1	84.7	50.3	62.0	59.1	87.9	73.2
**Route 3**	59.1	66.1	85.0	87.4	76.0	69.9	8.6	87.8	67.5
**All Routes**	64.3	73.0	85.1	83.3	64.2	64.9	43.9	87.7	70.8

**Table 8:** Decrease percentage of risky maneuvers on all routes for all vehicles (*V_1_ & V_2_*).

Percentage of Correct Alerts [%]										
Drivers	D_1_	D_2_	D_3_	D_4_	D_5_	D_6_	D_7_	D_8_	Avg.	Std. Dev.
**All Routes**	56.7	70.9	72.2	71.3	71.1	67.9	48.5	84.2	67.8	10.8
